# A pulmonary nodule mislocated in “dorsal” segment due to tri-lobed left lung

**DOI:** 10.3389/fsurg.2022.1069543

**Published:** 2023-01-06

**Authors:** Xiaohong Lyu, Yuan Xu, Yingzhi Qin, Dongjie Ma, Hongsheng Liu

**Affiliations:** ^1^ Department of Thoracic Surgery, Peking Union Medical College Hospital, Chinese Academy of Medical Science and Peking Union Medical College, Beijing, China; ^2^ Chinese Academy of Medical Science and Peking Union Medical College, Beijing, China

**Keywords:** lung cancer surgery, diagnosis, lung cancer, CT scan, computed tomography, chest, anatomy

## Abstract

**Background:**

The left lung has two lobes and one fissure, while the right lung has three lobes and two fissures. Accessory fissures are usually found in imaging examinations and autopsies; however, finding an actual accessory lobe is rare.

**Case presentation:**

In a lung nodule resection surgery, a 68-year-old male patient was found with three lobes and two fissures in his left lung. The lung nodule was misdiagnosed as being located in the lower lobe because the accessory fissure was misregarded as the oblique fissure. The lung nodule was found in the upper lobe, and this anatomical variation changed the surgical plan. The pathology of the lung nodule was granulomatous inflammation with caseous necrosis with the positive antacid stain. The patient was eventually diagnosed with tuberculosis.

**Literature review:**

Cases involving the lung accessory fissure and lung accessory lobe variants were reviewed. In 10 autopsy and dissection studies, the incidence of accessory fissure in the left lung was 13.5% (79/587, ranging from 2.7% to 50.0%), and in the right lung, it was 7.3% (42/575, ranging from 3.1% to 30.4%). The incidence of accessory lobes in the left lung was 2.0% (11/547, ranging from 0.0% to 7.4%), and in the right lung was 2.6% (14/539, ranging from 0.0% to 17.4%). The incidence of accessory fissures in bilateral lungs identified by chest x-ray or computed tomography ranged from 7.3% to 32.0%. Three surgical case reports inferred accessory lobes, including a left upper lobectomy, left lung transplantation, and an open thoracotomy.

**Conclusion:**

This is the first clinical case report that shows that lung accessory lobe caused the mislocation of a lung nodule. Therefore, radiologists and surgeons should be aware of the possibility of an accessory lobe in the lung.

## Introduction

The lungs are essential organs of respiration and are situated in the thoracic cavity on either side of the mediastinum. They are divided by fissures into lobes, facilitating movements of lobes toward one another. Usually, the left lung is divided by an oblique fissure into two lobes, namely the upper and lower lobes. The right lung is divided by oblique and horizontal fissures into the upper, middle, and lower lobes.

The spectacular advances in thoracic surgery in significant part date from the successful lobectomies performed during the early 1930s and from Graham's successful pneumonectomy for carcinoma of the lung in 1933 (1). If the surgeon's efforts were confined to the resection of a lobe or a whole lung, the importance of segmental anatomy was not fully appreciated. However, many factors still caused the misdiagnosis in segmental anatomy confirmation before surgery. The lung anatomical variations were one of the most frequently occurring causes of misdiagnosis.

Awareness of anatomical variations of lobes of the lung is essential because radiologists may misinterpret them on a chest x-ray (CXR) or a computed tomography (CT) scan. To our knowledge, there has been no report on the misdiagnosis before the lobectomy due to the anatomical variations. We herein report a 68-year-old male who was misdiagnosed with a pulmonary nodule located in the left dorsal segment (S6) before surgery. He was found to have a complete fissure splitting the upper lobe into two lobes, and the nodule was actually located in the apicoposterior segment (S1 + 2) as an accessory lobe.

## Case presentation

**Figure 1:**
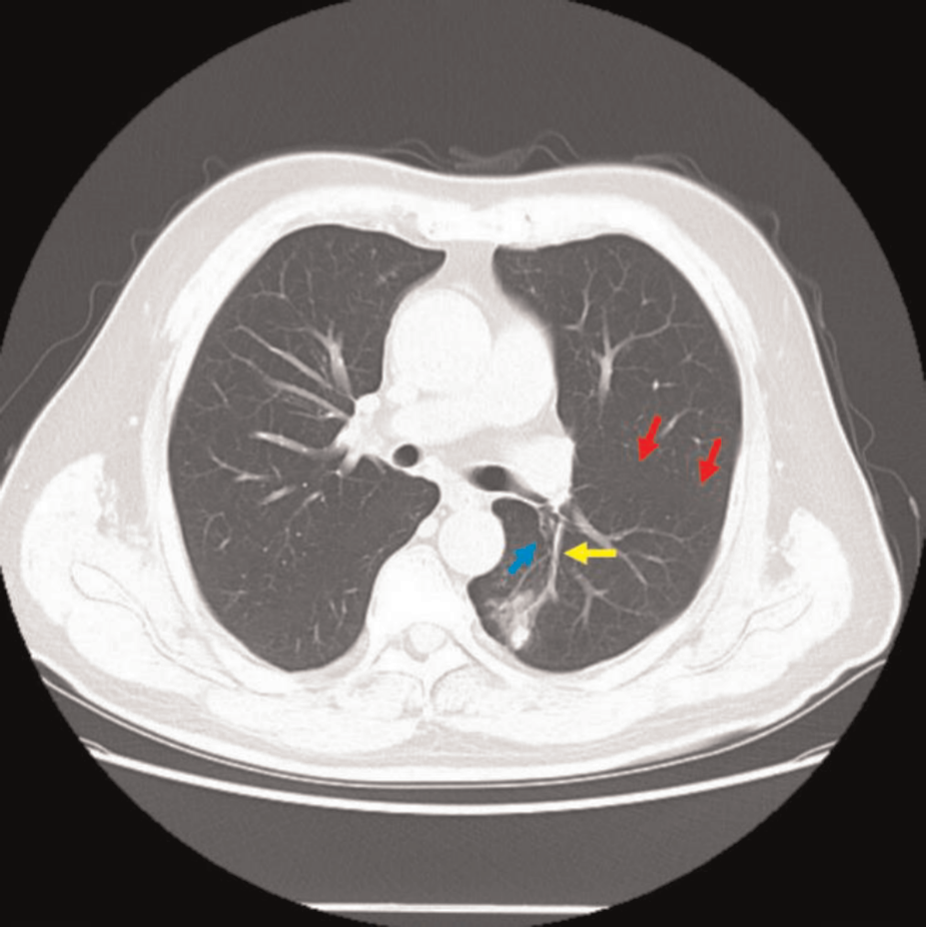
CT imaging of the patient before the surgery. The red arrow refers to the oblique fissure, the yellow arrow refers to the S6 artery, and the blue arrow refers to the S6 bronchus, regarded by radiologists and surgeons.

A 68-year-old male was discovered to have a pulmonary nodule in a CT scan for 4 years with no apparent symptoms. The latest CT scan (10/2021) indicated a blade-like hyperdense shadow (2.4 cm × 2.0 cm) in the lower-left lung (Figure 1), which had a higher density than before (06/2019). The positron emission tomography/CT (PET/CT) showed that the standard uptake value (SUV) max of the nodule was 8.1 and had high suggestive of malignancy. Both CT and PET/CT reported that the nodule was located in the left dorsal segment (S6) (Figure 1). Considering the possibility of high suggestive malignancy, it was planned for this patient to receive a video-assisted thoracic surgery (VATS) lobectomy of the left lower lobe.

**Figure 2:**
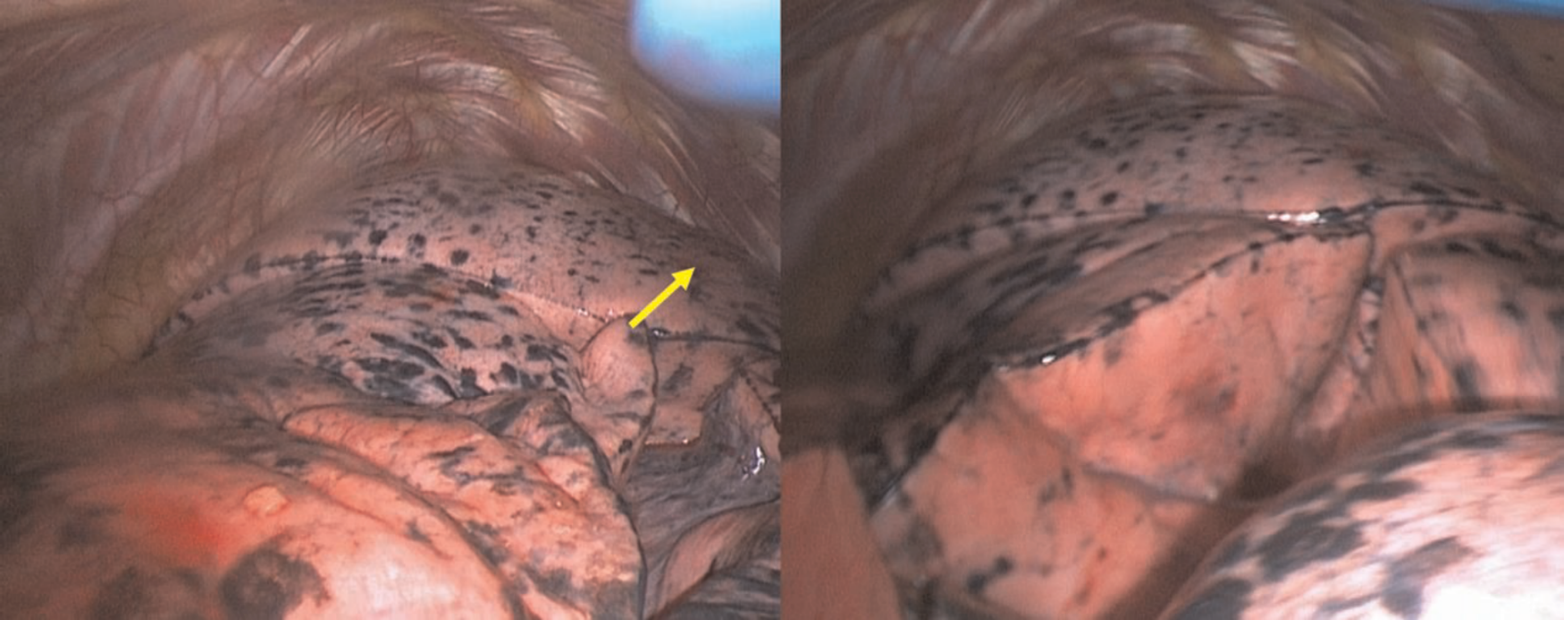
The three lobes of left lung photographed in the VATS. The yellow arrow refers to the lung nodule which cannot be found in the surface.

During intraoperative exploration, the patient was found to have a complete fissure splitting the upper lobe into two lobes (Figure 2). The accessory fissure completely splits the apicoposterior segment (S1 + 2) from the upper lobe. Therefore, there were three lobes in the left lung, including the S1 + 2 as an accessory lobe, the rest of the upper lobe, and the lower lobe. In addition, the target nodule was actually in the S1 + 2, not the S6 considered before surgery.

The surgery was changed to a segmentectomy of the S1 + 2. After the surgery, the patient recovered well and was discharged from the hospital after three days. The pathology of the lung nodule was granulomatous inflammation with caseous necrosis, with the positive antacid stain. The patient was eventually diagnosed with tuberculosis.

**Figure 3:**
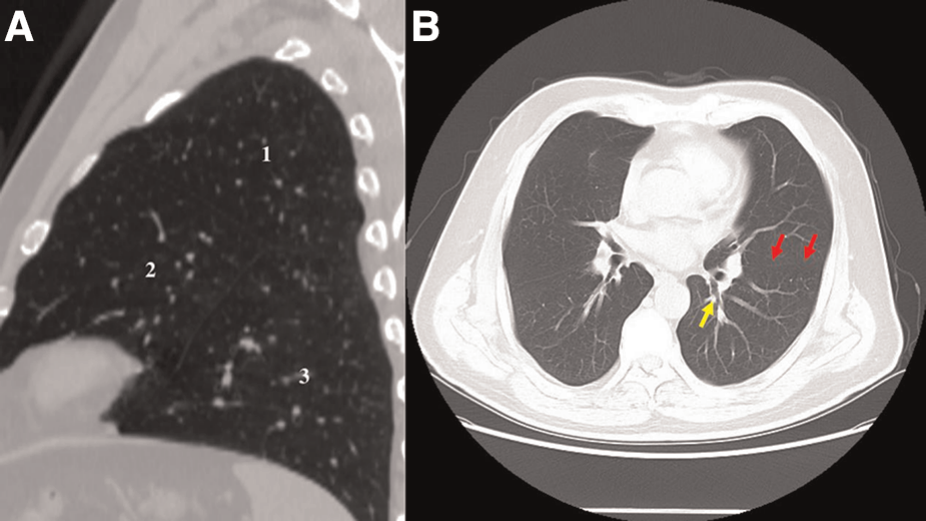
(**A**) CT image before the surgery shows the three lobes of the left lung: 1, accessory lobe (S1 + 2); 2, the rest upper lobe; 3, lower lobe. (**B**) CT image before the surgery. The red arrow refers to true oblique fissure. The yellow arrow refers to the true S6 bronchus.

**Figure 4:**
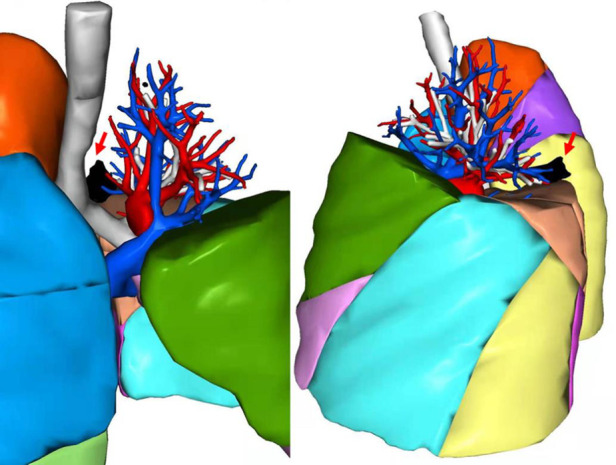
3D reconstruction of CT. Red arrows refers the lung nodule in black.

This misdiagnosis of location could be avoided, as the additional fissure, the real bronchus, and vessels of S1 + 2 and S6 were not tricky to identify in preoperative CT (Figure 3). Figure 4 shows a three-dimensional (3D) reconstruction of the lungs. The nodule was located in the S + 2, adjacent to the S6. A1 + 2a and A1 + 2b come from the posterior ascending artery, while A1 + 2c comes from the first trunk of the pulmonary artery.

## Literature review and discussion

Fissural anomalies include the presence of accessory fissures that develop to subdivide lobes or segments of the lung. The commonly described accessory fissures (also called supernumerary fissures) are the azygos fissure, superior accessory fissure, inferior accessory fissure, and left and right minor fissures (2–6). The most common accessory fissure described by anatomical studies (7–10) is the left minor fissure, which was first described by Boyden (11). Inferior accessory fissure (12) and left minor fissure (2) were the first and second most common accessory fissures detected by CT.

Grade classification of lung fissures, including accessory fissure proposed, by Craig and Walker (13) in 1997:

• Grade 1, complete fissure with entirely separate lobes.
• Grade 2, complete visceral cleft with parenchymal fusion at the base of the fissure.
• Grade 3, visceral cleft is evident for part of the fissure.
• Grade 4, complete fusion of the lobes with no evident fissural line.

Only when the accessory fissure is at Grade 1, which means a complete fissure with entirely separate lobes, does the accessory fissure demarcate the accessory lobe.

Accessory fissures are commonly identified incidentally during autopsy, dissection, and imaging examinations performed on the chest, including CXR and CT. Their presence may be of clinical importance both surgically and radiologically (14). Accessory fissures can be mistakenly confused with areas of linear atelectasis, pleural scars, pleural effusion, or walls of bullae (15, 16). The presence of a superior accessory fissure of the lower lobe correlates with characteristic patterns of atelectasis and pleural fluid collection on conventional radiographs (17). In the presence of extra lobes, the CT scan showed a significant increase in the size of the mediastinum around the trachea (6). Familiarization with accessory fissures is essential because they may be mistaken for a disease condition.

Previous studies showed a wide variation in accessory fissures and lobes occurrence (2, 15, 18). Therefore, it is necessary to review case reports of accessory fissures and lobes in both lungs in the process of autopsy, imaging examination, and surgery. The incidences of accessory fissure and accessory lobe in autopsy or dissection were reviewed in Table 1, in imaging examination as Table 2, and in lung resection surgery as Table 3.

**Table 1:** The literature reporting lung with accessory fissures and three lobes in autopsy and dissection.

Author-year	Country	Sample size	Left lung		Right lung	
			Accessory fissure (%, *k*/*n*)	Three lobes (%, *k*/*n*)	Accessory fissure (%, *k*/*n*)	Four lobes (%, *k*/*n*)
Halagatti-2020 (22)	India	Left 37 Right 37	2.7% (1/37)	0.0% (0/37)	5.4% (2/37)	0.0% (0/37)
Kc-2018 (7)	Nepal	Left 27 Right 23	33.3% (9/27)	7.4% (2/27)	30.4% (7/23)	17.4% (4/23)
Unver Dogan-2015 (23)	Turkey	Left 210 Right 210	5.7% (12/210)	1.0% (2/210)	3.8% (8/210)	1.9% (4/210)
Magadum-2015 (10)	India	Left 40 Right 40	7.5% (3/40)	0.0% (0/40)	7.5% (3/40)	0.0% (0/40)
Quadros-2014 (9)	India	Left 40 Right 36	22.5% (9/40)	NM^a^	13.9% (5/36)	NM
George-2014 (24)	India	Left 73 Right 65	2.7% (2/73)	2.7% (2/73)	4.6% (3/65)	4.6% (3/65)
Esomonu-2013 (25)	Nigeria	1 case report	1	1	1	1
Murlimanju-2012 (26)	India	Left 28 Right 32	10.7% (3/28)	3.6% (1/28)	3.1% (1/32)	3.1% (1/32)
Nene-2011 (8)	India	Left 50 Right 50	50.0% (25/50)	0.0% (0/50)	18% (9/50)	2.0% (1/50)
Gesase-2006 (27)	Tanzania	Left 51 Right 51	21.6% (11/51)	2.9% (3/51)	3.9% (2/51)	0.0% (0/51)
Meenakshi-2004 (16)	India	Left 30 Right 30	10.0% (3/30)	0.0% (0/30)	3.3% (1/30)	0.0% (0/30)
**Average**	** **	** **	**13.5%** **(79/587)**	**2.0%** **(11/547)**	**7.3%** **(42/575)**	**2.6%** **(14/539)**

^a^ NM, not mentioned.

**Table 2:** The literature reporting lung with accessory fissures and three lobes in radiological procedure including CXR and (HR)CT.

Author-year	Country	Radiological procedure	Sample size	Total accessory fissure	Left lung		Right lung	
					Accessory fissure (%, *k*/*n*)	Three lobes (%, *k*/*n*)	Accessory fissure (%, *k*/*n*)	Four lobes (%, *k*/*n*)
Manjunath-2021 (12)	India	CT	560	7.3% (41/560)	3.9% (22/560)	0	3.4% (19/560)	0
Aziz-2004 (18)	Japan	HRCT	622	20.3% (126/622)	10.6% (66/622)	NM^b^	9.6% (60/622)	NM
Ariyurek-2001 (2)	Turkey	HRCT	186	32.0% (59/186)	NM	NM	NM	NM
Takasugi-1989 (5)	USA	CXR	1 Case report	0	0	0	0	0
		CT	1 Case report	1	1	1	0	0
Austin-1986 (3)	USA	CXR	2,000	NM	1.6% (32/2,000) ^a^	NM	NM	NM
Godwin-1984 (15)	USA	CXR	50	12.0% (6/50)	NM	NM	NM	NM
		CT	50	18.0% (9/50)	NM	NM	NM	NM

^a^ This frequency only referred to the left minor fissures in left lung, instead of all the accessory fissures.

^b^ NM, not mentioned.

**Table 3:** The literature reporting lung with accessory fissures and three lobes in lung resection surgery case report.

Author-year	Country	Surgery	Sample size	Left lung		Right lung	
				Accessory fissure (%, *k*/*n*)	Three lobes (%, *k*/*n*)	Accessory fissure (%, *k*/*n*)	Four lobes (%, *k*/*n*)
Wells-1960 (19)	India	Left upper lobectomy	1 Case report	1	1	0	0
Jacob-2019 (20)	USA	Left Lung Transplantation of the donor	1 Case report	1	1	0	0
Mehrabi-2021 (21)	Iran	open thoracotomy	1 Case report	2 Accessory fissures	4 Lobes	0	0

In ten autopsy and dissection studies from five countries (Table 1), the incidence of accessory fissure in the left lung was 13.5% (79/587, ranging from 2.7% to 50.0%), and in the right lung, it was 7.3% (42/575, ranging from 3.1% to 30.4%). The incidence of accessory lobes in the left lung was 2.0% (11/547, ranging from 0.0% to 7.4%), and in the right lung, it was 2.6% (14/539, ranging from 0.0% to 17.4%). According to the result of the case review, the variation that there were three lobes in the left lung was rare, and the incidence was about 2.0% in the previous studies.

In five radiological studies (Table 2), the incidence of accessory fissures in bilateral lungs found by CXR or CT ranged from 7.3% to 32.0%. There were 20.3% (126/622) (18) to 32.0% (59/186) (2) patients detected with accessory fissures in high-resolution CT (HRCT). 7.3% (41/560) of the patients were detected with an accessory fissure by CT (12). Therefore, the screening effect of CT for accessory fissures might be lower than that of HRCT in the previous study. The effect of CXR to detect accessory fissures might also be worse than CT or HRCT (5, 15).

The accessory lobes discovered in surgeries were reported in three case reports, including a left upper lobectomy (19) in 1960, a left lung transplantation (20) in 2019, and an open thoracotomy (21) in 2021. In history, when the doctors reported meeting the true tri-lobes in the left lung for the first time all around the world (19), the first thought that came to their mind was whether this patient had a “situ inversus.” Nevertheless, when the stomach and heart were in their normal position, as seen in the plain film of the chest, they confirmed that the patient had tri-lobes in the left lung. Moreover, in a left lung transplantation case report (20), the left lung from the donor was recognized with tri-lobes until the surgery team received the lung. In another open thoracotomy case report, the surgeon team (21) reported a bilateral primary spontaneous pneumothorax case that underwent talc slurry and bleomycin pleurodesis at the right and left sides retrospectively, complicated with left-sided recurrent spontaneous pneumothorax. The patient underwent open thoracotomy and was accidentally found to have four lobes and three fissures in the left lung. The knowledge of variant fissures is fundamental, especially in the pre-operative planning of lung surgery.

How and why accessory fissures occurs in the lung is not known. The fissures of the lungs are embryologically separating the bronchopulmonary segments, which later on persist in the interlobar planes of the fully developed lung. All the variations noted in lobulation and fissures in both lungs might result from altered pulmonary development. In humans and other species, including dogs, cats, bovines, horses, et al., the accessory lobes were reported in the previous studies (21). Generally, they are considered to be incidental findings that are not associated with lung symptoms.

## Conclusion

To our knowledge, this is the first report of a mislocated lung nodule because of a left lung with three lobes. The presence of this accessory fissure and accessory lobe changed the surgical plan. We reviewed the literature inferring accessory fissures in autopsy, dissection, imaging examination, and surgery. The incidence of three lobes in the left lung was about 2.0%. The radiologists and surgeons should be aware of the accessory lobe possibility.
